# Anderson localization in synthetic photonic lattices

**DOI:** 10.1038/s41598-017-04059-z

**Published:** 2017-06-27

**Authors:** Ilya D. Vatnik, Alexey Tikan, Georgy Onishchukov, Dmitry V. Churkin, Andrey A. Sukhorukov

**Affiliations:** 1 Novosibirsk State University, 2 Pirogova str., Novosibirsk, 630090 Russia; 2 Institute of Automation and Electrometry SB RAS, Novosibirsk, 630090 Russia; 3 Institute of Microwaves and Photonics, (LHFT), Friedrich-Alexander University Erlangen-Nürnberg, Erlangen, 91058 Germany; 4 Nonlinear Physics Centre, Research School of Physics and Engineering, Australian National University, Canberra, ACT 2601 Australia

## Abstract

Synthetic photonic lattices provide unique capabilities to realize theoretical concepts emerging in different fields of wave physics via the utilization of powerful photonic technologies. Here we observe experimentally Anderson localization for optical pulses in time domain, using a photonic mesh lattice composed of coupled fiber loops. We introduce a random potential through programmed electro-optic pulse phase modulation, and identify the localization features associated with varying degree of disorder. Furthermore, we present a practical approach to control the band-gap width in photonic lattices by varying the coupling between the fiber loops, and reveal that the strongest degree of localization is limited and increases in lattices with wider band-gaps. Importantly, this opens a possibility to enhance or reduce the effect of disorder and associated localization of optical pulses.

## Introduction

An intriguing concept of wave mechanics is Anderson localization, a phenomenon firstly formulated for electrons in crystals with defects^1^. The theory predicted that static, or time-independent, disorder can lead to complete localization of wavefunctions for non-interacting particles. Optics offers a fruitful framework to achieve this regime, as it’s easier to preserve coherence in optical systems, and photon interactions can be vanishingly small at low light intensities. Indeed, Anderson localization was observed experimentally for photons in lattices and photonic-crystal structures^2–4^, see a review in ref. 5. Beyond the fundamental importance recognized by Nobel prize for the original discovery^1^, the Anderson localization can find multiple applications including image transmission in disordered fibers^6–8^. Whereas optical localization was initially observed in space^2, 3^, its implementation in time can open new opportunities for optical pulse manipulation, and this is the focus of current work.

It was demonstrated that synthetic photonic lattices (SPL) for optical pulses in time domain can be realized in coupled optical ring resonators with different path-lengths^9–12^, building on the time-multiplexing concepts originally developed for photon detectors^13, 14^. A number of important effects have been demonstrated in SPLs, including random walks of single particles^11^, Bloch oscillations and unidirectional invisibility associated with parity-time symmetry^12, 15^, scattering on defect states^16^, and diametric drive acceleration^17^. Furthermore, SPLs are naturally suitable for observation of Anderson localization, since any degree of disorder can be introduced through programmable electro-optic phase-modulation of individual propagating pulses at specific time slots of the lattice. First observation of Anderson localization in a system conceptually similar to an SPL was reported in refs 9, 10. In this implementation, a set of two polarizations effectively play a role of two different loops with different propagation time. However, only the regime of strong disorder was realized^10^, as large random phase shifts in comparison with the phase acquired by a pulse for one roundtrip were applied. Whereas Anderson localization can occur for arbitrarily weak disorder in one-dimensional potentials^18, 19^, this regime remained unexplored in SPLs.

In this paper, we present results of comprehensive experimental and numerical investigation of the effect of disorder on light pulses in synthetic photonic lattice composed of two fiber loops. Our results numerically confirm the onset of Anderson localization even at weak disorder, and we describe the localization features for different disorder strengths. Furthermore, we identify a practical approach to control photonic band-gap width by varying the coupling between the fiber loops, and show that this allows one to enhance or reduce localization, since the strongest degree of localization is limited and increases in lattices with wider band-gaps.

## Results

### Mesh photonic lattices with disorder

Following refs 11, 12, we consider a synthetic photonic lattice formed by two fiber loops of different lengths *L* and *L* + Δ*L* connected by a fiber coupler. Details of the experimental setup are provided in Methods and Supplementary Information section 1. Optical losses in the loops are precisely compensated by amplifiers. Each roundtrip of a pulse over a loop corresponds to a ‘time’ or ‘slow’ coordinate of a spatial mesh lattice, and is characterized by discrete number *m*. A light pulse changes its position in space of *n*, as roundtrip times in two loops are slightly different. The time delay between pulses (position number *n*), appearing at a photodiode coupling to the short loop, corresponds to ‘space’ or ‘fast’ coordinate of a spatial mesh lattice.

**Figure 1:**
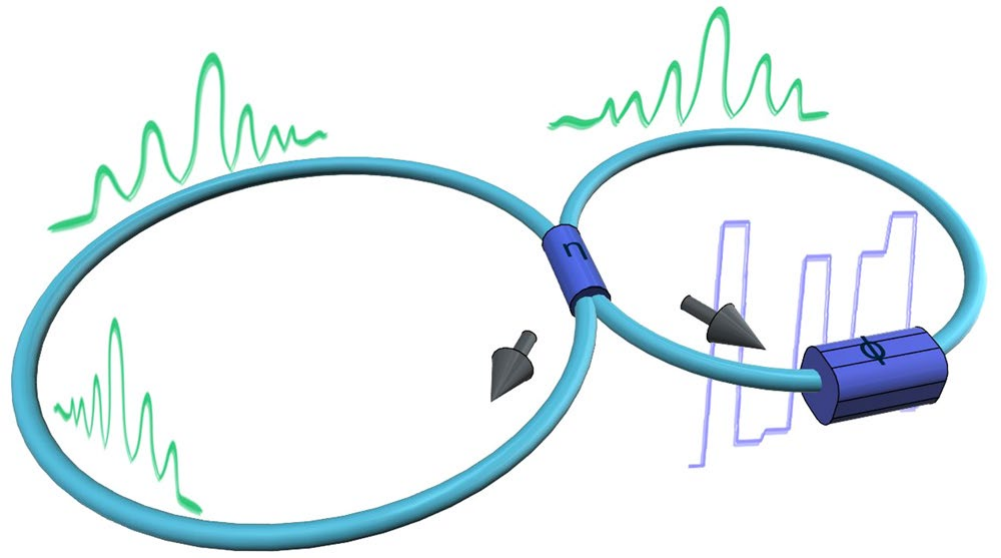
The scheme of the experimental realization of a synthetic mesh photonic lattice based on fiber loops with random potential.

The system is described by a set of equations for amplitudes of light pulses placed at ‘space’ position *n* in short (${U}_{n}^{m}$) and long (${V}_{n}^{m}$) loops at the *m*-th roundtrip^11, 12, 16, 17^:

1 $$\begin{array}{rcl}{U}_{n}^{m+1} & = & \exp (i{\varphi }_{n}^{m})\,[\,\cos (\eta ){U}_{n+1}^{m}+i\,\sin (\eta ){V}_{n+1}^{m}],\\ {V}_{n}^{m+1} & = & \cos (\eta ){V}_{n-1}^{m}+i\,\sin (\eta ){U}_{n-1}^{m},\end{array}$$

where ${\varphi }_{n}^{m}$ are phase shifts introduced by an electro-optical modulator placed inside the short loop. *η* defines the coupling ratio of the splitter connecting the loops, and in experiments we use a symmetrical 50:50 splitter corresponding to *η* = *π*/4 (Fig. 1).

## Discussion

We study the effect of disorder on a single pulse coupled into the long loop at the input. This corresponds to the initial conditions ${V}_{n=0}^{0}=1$, ${V}_{n\ne 0}^{0}=0$, and ${U}_{n}^{0}=0$. If there is no potential introduced in the system (${\varphi }_{n}=0$), we observe well-known ballistic expansion, which resembles quantum walk of a single particle^11^. In this regime, the width of a pulse chain grows proportionally to the number of roundtrips. Applying the phase shift *ϕ*_*n*_ which is randomly distributed over *n* (i.e. the phase shifts are transversely uncorrelated) and constant over *m*, the optical analogue of a random potential can be created.

**Figure 2:**
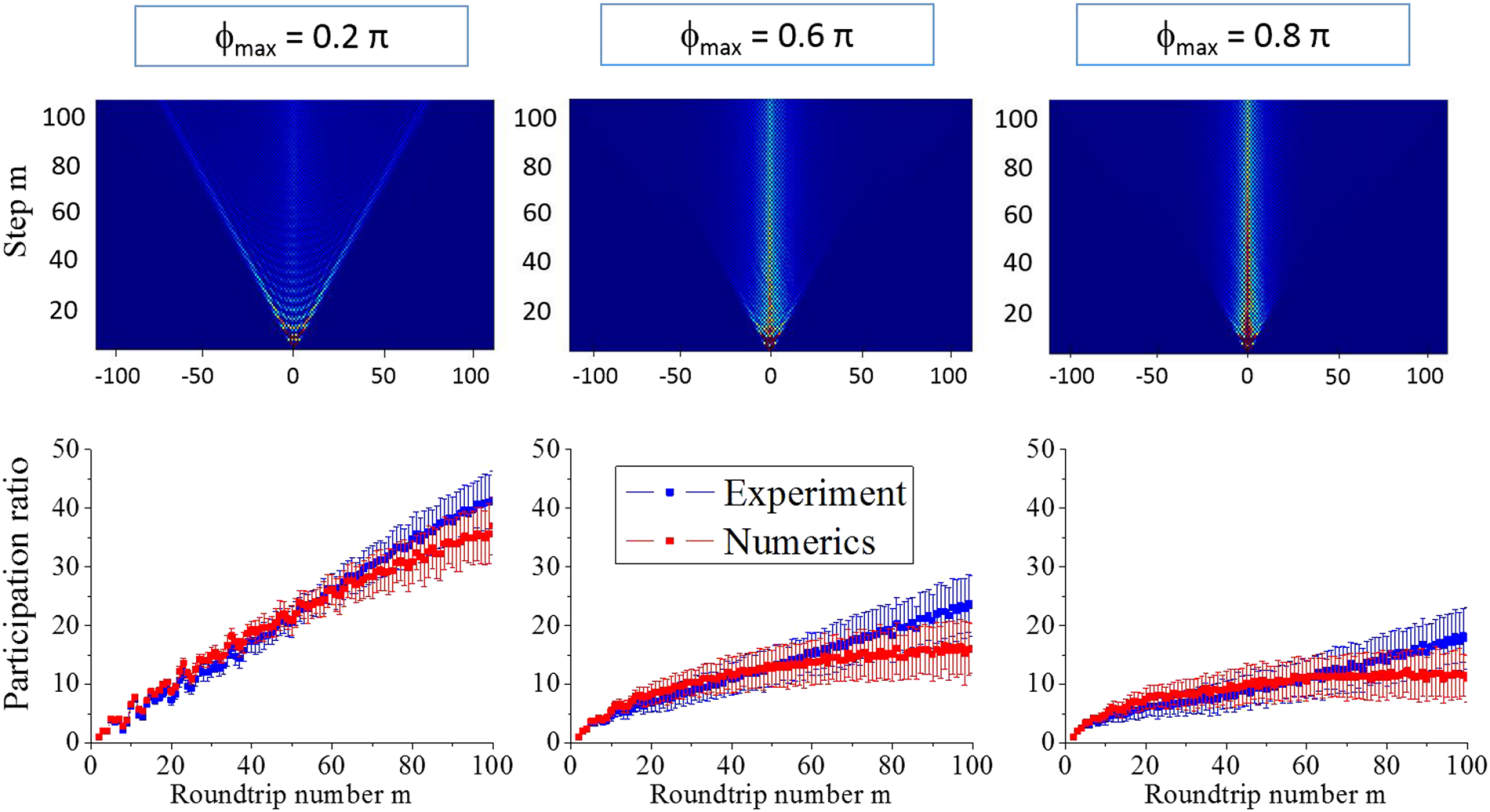
Upper panel: Experimental pulse evolution in synthetic photonic lattices with different strengths of randomly distributed potential as indicated by labels. Lower panel: corresponding evolution of the participation ratio determined from experimental data and numerical simulations. All data is averaged over 100 realizations and the variance of the participation ratio is calculated.

We gradually increase the strength of the potential *ϕ*_max_, where *ϕ*_*n*_ is randomly distributed in the interval (0, *ϕ*_max_), to study the onset of Anderson localization. For each level of disorder *ϕ*_max_ we carried out a set of 100 experiments, using a different realization of the potential in each experiment within the set. A single pulse was launched with the same temporal position (*n* = 0) into the long loop. Then, the ensemble-averaged values, including the average intensities $\langle {|{U}_{n}^{m}|}^{2}\rangle$ in the short loop, were extracted, see the top row in Fig. 2. The whole procedure was repeated for different *ϕ*_max_ values.

To quantify the process of localization, we analyze the participation ratio $P(m)={({\sum }_{n}{|{U}_{n}^{m}|}^{2})}^{2}/{\sum }_{n}\,{|{U}_{n}^{m}|}^{4}$ ^2^. The broader the distribution of a wavepacket ${U}_{n}^{m}$ at the roundtrip *m*, the larger is *P*(*m*). Due to the fact that we study the optical lattice realized in time domain, the value of participation ratio corresponds to the number of pulses in a chain propagating inside the loops. We present the experimental participation ratio and its variance over the ensemble in the bottom row of Fig. 2.

**Figure 3:**
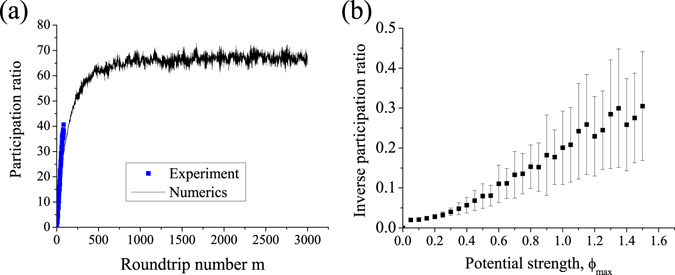
(**a**) Participation ratio calculated for the case of a weak potential realization with *ϕ*_max_ = 0.2*π*. (**b**) Inverse participation ratio, numerically derived and averaged over 100 realizations of random potential with different strengths for *m* = 120.

For each particular realization of the phase distribution *ϕ*_*n*_, we perform a comparison of experimental data with theoretical predictions based on Eq. (1). The phase distributions used for calculations were the same with those applied to the phase modulator in the experiment. Numerically calculated dependencies of participation ratio *P*(*m*) clearly confirm the presence of localization, see the bottom row of Fig. 2. We observe a good agreement between experimental results and numerics up to *m* = 80 roundtrips, although discrepancies appear between the predicted and observed participation ratios after longer propagation times, see Fig. 2(b). The positive bias for experimentally determined *P*(*m*) at large *m* is due to a gradual rise of optical noise in the system occurring after each roundtrip (see Supplementary Information section 1 for details), which efficiently increases the participation ratio. We extrapolate the results for larger values of *m* using numerical simulations, and confirm the onset of localization at $m\simeq 800$ in case of relatively weak random potential *ϕ*_max_ = 0.2*π*, see Fig. 3(a). Another confirmation of the effect of Anderson localization can be found by the inverse participation ratio at a fixed time step *m* = 100, calculated over an ensemble of realizations of disorder, and observe its increase as the stronger random potential is applied, see Fig. 3(b). Note that the fluctuations of inverse participation ratio *δ*(1/*P*) (*ϕ*_max_) increase with *ϕ*_max_ as well, which is an additional indication of the localization^2^.

**Figure 4:**
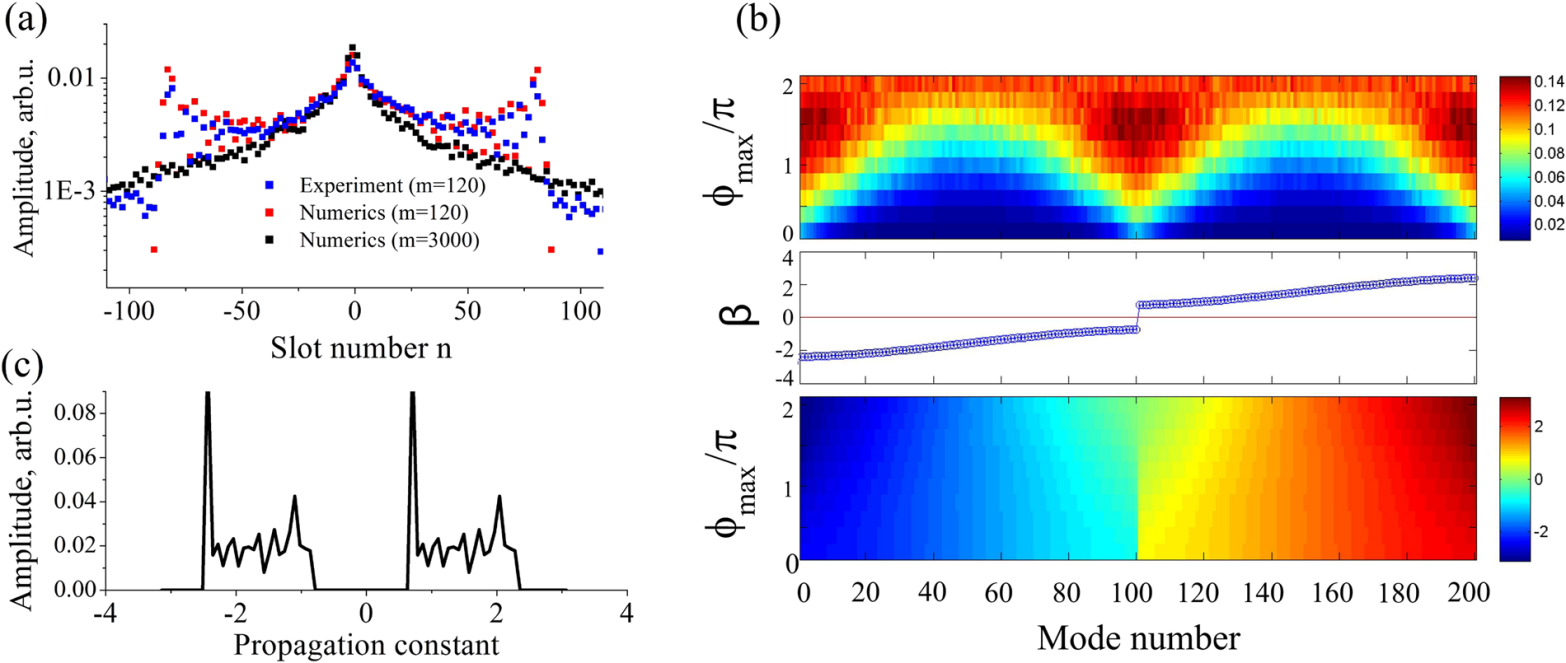
(**a**) Amplitudes of the pulse sequence propagating through the SPL with random potential strength *ϕ*_max_ = 0.2*π*, in experiment and numerical simulations. Data is averaged over 100 different random realizations. (**b**) Calculated inverse participation ratio for eigenmodes (upper panel), and the corresponding propagation constants *β* (lower panel) vs. the disorder strength (*ϕ*_max_). Middle panel shows the band structure for a trivial potential with *ϕ*_max_ = 0. (**c**) An eigenmode excitation spectrum for a single realization of a SPL with random potential of strength *ϕ*_max_ = 0.2*π*, excited by a single pulse.

We also observe a close fit of the experimentally measured and calculated intensity profiles up to *m* = 120, see blue and red squares in Fig. 4(a), respectively. To emphasize the localization process, we calculate numerically the intensity profile at *m* = 3000 and find it to have a typical form for Anderson localization with exponential tails, see Fig. 4(a), black squares.

To get an insight into the localization process, we simulate numerically the structure of eigenmodes. The eigenmode solutions have the form ${U}_{n}^{m}={U}_{n}\,\exp (im\beta )$, ${V}_{n}^{m}={V}_{n}\,\exp (im\beta )$, where *β* is the propagation constant. Such modes form a complete set of solutions for a potential which is random along *n* coordinate and constant along *m*. By substituting these expressions into Eq. (1), we obtain a set of 2*N* coupled linear equations, and then determine their eigenmodes numerically (see Supplementary Section 2 for details). Here *N* is a number of pulses that can be placed in loops of a given length simultaneously. In experiments, *N* is defined by the ratio of the width of a time slot and the roundtrip time, *N* = *L*/Δ*L*.

The calculated mode spectra for synthetic photonic lattices with different levels of disorder are presented in Fig. 4(b), lower panel. If there is no phase potential (${\varphi }_{{\rm{\max }}}\equiv 0$), the mode spectrum can be found analytically in the limit of infinite lattice size,

2 $${\beta }_{\pm }=\pm {\cos }^{-1}\,[\,\cos (K)\,\cos (\eta )],$$

where *K* is the wavenumber in the *n* direction. The spectrum consists of two bands separated by a band-gap, see the middle panel in Fig. 4(b).

At small disorder, the mode spectrum is almost the same as for a homogeneous lattice. The stronger the random potential is, the narrower the bandgap becomes, and for maximum disorder the band-gap width approaches zero. But even for a weak random potential, localized modes immediately appear. The strongest localized modes are located at the edge of the band-gap, as indicated by maxima of the inverse participation ratio 1/*P* for mode numbers ~0, 100, 200 in Fig. 4(b), upper panel. This is analogous to the features of Anderson localization in waveguide arrays^3^. A structure of typical localized modes in our system is discussed in the Supplementary Section 3.

We also simulate the mode excitation spectrum corresponding to our initial conditions, when a single pulse is coupled into the long loop at the input (we briefly discuss the features of multi-pulse excitation in the Supplementary Section 4). We find that the excitation spectrum primarily consist of the well-localized modes at the edge of the bandgap, see a representative example in Fig. 4(c). Since the propagation is linear and the phase potential is fixed for each disorder realization, the excitation spectrum is preserved during the evolution.

**Figure 5:**
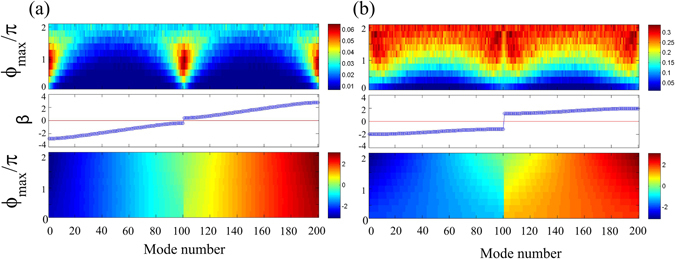
Inverse participation ratio for modes (upper panels) and the corresponding propagation constants *β* (lower panels) vs. the disorder strength (*ϕ*_max_) for different coupling ratios: (**a**) *η* = *π*/8 and (**b**) *η* = 3*π*/8. Middle panels show the mode band structure for a trivial potential with *ϕ*_max_ = 0.

Synthetic photonic lattices allow one to change the coupling strength between fibre loops. We study the effect of the coupling strength (*η*) on localization. We note that in the absence of disorder, according to Eq. (2), the width of transmission bands is (*π* − 2*η*) and the gap width is 2*η*. Whereas a symmetrical 50:50 splitter (*η* = *π*/4) was used in our experiments, we also perform numerical simulations for the coupling ratios *η* = *π*/8 and 3*π*/8. In agreement with analytical predictions, the gap width increases for larger *η*, see the middle and lower panels in Fig. 5(a,b). Accordingly, the degree of Anderson localization also increases, corresponding to larger inverse participation ratio values, cf. the upper panels in Fig. 5(a,b).

For a small fiber loop coupling (*η* = *π*/8), the localization strength depends nontrivially on the disorder [Fig. 5(a)]. The maximum localization is observed at the band edges for ${\varphi }_{{\rm{\max }}}\mathop{ < }\limits_{ \tilde {}}1.5\pi$. This agrees with the predictions made for photonic crystals^20^, that the smallest localized mode size near a band-edge is defined by the flatness of the dispersion curve, and modes should get more localized for higher disorder. However for stronger phase modulation the band-gap closes due to disorder. In this regime, localization gets weaker at the former band-edge locations, since the effective dispersion slope becomes larger. As a result, all modes exhibit similar yet relatively weak degree of localization, which is lower than for band-edge modes at smaller disorder. In contrast, for larger fiber loop coupling (*η* = 3*π*/8), the band-gap never closes fully, and a degree of localization grows gradually for increasing disorder [Fig. 5(b)].

## Conclusions

To conclude, we have simulated numerically and demonstrated experimentally Anderson localization of optical pulses in synthetic photonic mesh lattices composed of coupled fiber loops. We find that localized modes arise even for weak disorder in full agreement with the theory of Anderson localization in one-dimensional systems. Importantly, we reveal that mesh lattices can be designed to enhance or reduce localization by varying the coupling between the fiber loops. Such coupling tunes the photonic band-gap, and we find that the strongest degree of localization is limited and increases in lattices with wider band-gaps, for the same level of disorder. This mechanism of localization control can find application in optical fiber systems, and can also be realized in other optical systems based on the generic nature of wave phenomena. Furthermore, our results suggest new implications for quantum random walk emulators.

## Methods

We used a pair of 5 km non-zero dispersion-shifted telecommunication fiber spools to form two loops of the synthetic photonic lattice with the 48 m difference in length. Pulse width, formed by direct current modulation of a FBG-stabilized diode laser, was set to 100 ns. Optical losses of all elements were compensated using semiconductor optical amplifiers together with optical filters to suppress amplified spontaneous emission. Spontaneous emission from amplifiers in backward direction was dumped by optical isolators. In addition, we increase losses in the loops considerably before each measurement by means of acousto-optic modulators and set it back at the very moment of the launching the first pulse into the system. This approach allows us to suppress noise circulating in the loops. More discussion on experimental details and noise effects are provided in Supplementary Section 1. Pulse polarization was controlled by several polarization controllers and monitored using a polarization beam splitter. To create a time-domain analog of an optical potential, the electro-optical phase modulator (EOM) is added in the shorter loop driven with an arbitrary wave generator. The generator signal is a specially designed pulse pattern allowing us to generate arbitrary phase distributions along the fast coordinate but constant along the slow coordinate. We included a photodiode detecting a pulse train in the short loop, and the measured signal was further processed to characterize the states of the synthetic photonic lattice.

## Electronic supplementary material

Supplementary Information
