# Supplementary material for: Anderson localization in synthetic photonic lattices
Source: Sci Rep. 2017 Jun 27;7:4301. doi: 10.1038/s41598-017-04059-z (PMC5487357; doi:10.1038/s41598-017-04059-z)
Supplement: Supplementary file 1 — Supplementary Information [file 41598_2017_4059_MOESM1_ESM.pdf]

Anderson localization in synthetic photonic lattices

Ilya D. Vatnik[1], Alexey Tikan[1], Georgy Onishchukov[2], Dmitry V. Churkin[1,*], and Andrey A. Sukhorukov[3]

[1]Novosibirsk State University, 2 Pirogova str., Novosibirsk, 630090, Russia

[2]Institute of Microwaves and Photonics, University of Erlangen, 91058, Germany

[3]Nonlinear Physics Centre, Research School of Physics and Engineering, Australian National University, Canberra, ACT 2601, Australia

*churkin@nsu.ru

1. Experimental setup and limitations on a number of roundtrips

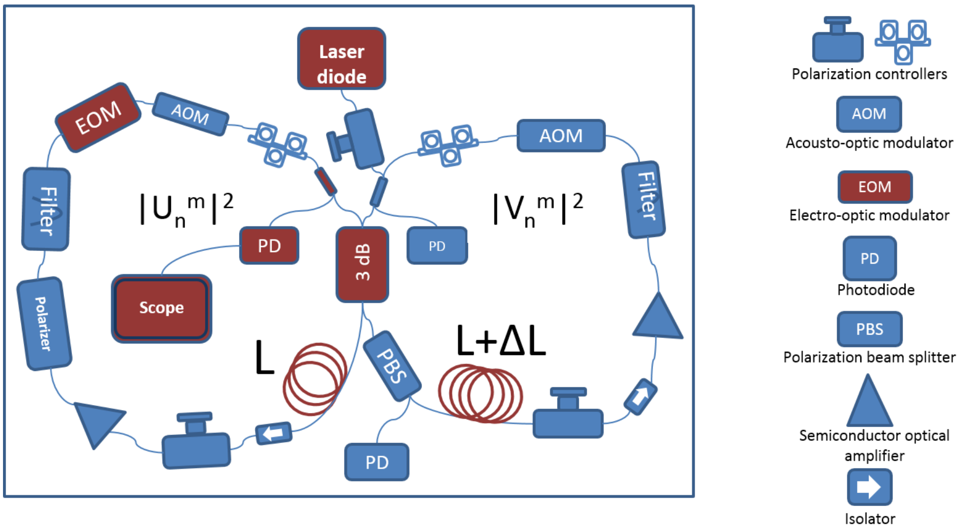

Figure S1. Detailed experimental setup

The synthetic photonic lattice is formed by two fiber loops of different length L and L+ΔL connected by a fiber coupler (Figure S1). A pulse from directly modulated FBG-stabilized diode laser is launched into the system by a coupler and then divided into two replicas (in each of two loops). Due to a difference in the loop lengths, these pulses multiplex into chains of pulses, which interfere with each other from roundtrip to roundtrip. To compensate all the losses in the system, semiconductor optical amplifiers are used together with optical filters to suppress amplified spontaneous emission. To exclude any contribution from spontaneous emission going in the backward direction, isolators are used. Pulse polarization shifts due to fiber imperfections, and birefringence is compensated by several polarization controllers. Polarization state is monitored using a polarization beam splitter. To create a time-domain analog of an optical potential, the electro-optical phase modulator (EOM) is added in the shorter loop driven with an arbitrary wave generator. Two photodiodes are used to measure time traces from both short and long loops.

The synthetic photonic lattice has the capability of observing the packet evolution over larger effective propagation distance comparing with waveguide arrays. However, there are some limitations due to different noise sources. One of the major problems is due to noise of optical amplifiers. Indeed, since losses within the loops are exactly compensated by the amplifiers, a noise signal caused by the spontaneous emission of the laser diode and the semiconductor amplifiers grows in the system from roundtrip to roundtrip. The magnitude of the spontaneous signal becomes significant with a large number of round trips and ultimately prevails over the useful signal.

To deal with this, the following method is used. We perform suppression of optical signals by means of acoustooptic modulators that increase the losses in both loops by a large amount (about 13 dB) for a long time before the start of each experiment. After we set the losses to normal values to start the experiment, immediately the spontaneous noise starts to grow, and it becomes comparable with the useful signal after 100-150 roundtrips.

The increase in total number of roundtrips can be achieved by using amplifiers with lower noise, see Ref. [1].

2. Theoretical calculation of eigenmodes in presence of disorder

We seek eigenmode solutions of the model Eq. (1), assuming that the modulator phase is randomly distributed along the coordinate n and invariable along the coordinate m. Then, the eigenmodes can be written in the form $u_{n,m}=U_n\exp(im\beta)$ - for a short loop, $v_{n,m}=V_n\exp(im\beta)$ - for a long loop, where β is propagation constant. Substituting the expressions in Eq. (1), we obtain the following coupled equations:

$$\begin{pmatrix} VV & VU \\ UV & UU \end{pmatrix} * \begin{pmatrix} v_1^m \\ \vdots \\ v_N^m \\ u_1^m \\ \vdots \\ u_N^m \end{pmatrix} = \begin{pmatrix} v_1^{m+1} \\ \vdots \\ v_N^{m+1} \\ u_1^{m+1} \\ \vdots \\ u_N^{m+1} \end{pmatrix} = \lambda * \begin{pmatrix} v_1^m \\ \vdots \\ v_N^m \\ u_1^m \\ \vdots \\ u_N^m \end{pmatrix} \tag{S1}$$

where the propagation constant is expressed as $\beta=\log(\lambda)/i$, and the matrices VV, VU, UV and UU are defined as follows considering periodic boundary conditions:

$$VV = \begin{pmatrix} 0 & 0 & 0 & \cdots & \cos(\eta) \\ \cos(\eta) & 0 & 0 & \cdots & 0 \\ 0 & \cos(\eta) & 0 & \cdots & 0 \\ \vdots & \vdots & \ddots & \ddots & \vdots \\ 0 & \cdots & 0 & \cos(\eta) & 0 \end{pmatrix}$$

$$VU=\begin{pmatrix} 0 & 0 & 0 & \cdots & i\sin(\eta) \\ i\sin(\eta) & 0 & 0 & \cdots & 0 \\ 0 & i\sin(\eta) & 0 & \cdots & 0 \\ \vdots & \vdots & \ddots & \ddots & \vdots \\ 0 & \cdots & 0 & i\sin(\eta) & 0 \end{pmatrix}$$

$$UV=\begin{pmatrix} 0 & i\sin(\eta)e^{i\phi_1} & 0 & \cdots & 0 \\ 0 & 0 & i\sin(\eta)e^{i\phi_2} & \cdots & 0 \\ \vdots & \vdots & \vdots & \ddots & \vdots \\ 0 & 0 & 0 & \cdots & i\sin(\eta)e^{i\phi_{N-1}} \\ i\sin(\eta)e^{i\phi_N} & 0 & \cdots & 0 & 0 \end{pmatrix}$$

$$UU=\begin{pmatrix} 0 & \cos(\eta)e^{i\phi_1} & 0 & \cdots & 0 \\ 0 & 0 & \cos(\eta)e^{i\phi_2} & \cdots & 0 \\ \vdots & \vdots & \vdots & \ddots & \vdots \\ 0 & 0 & 0 & \cdots & \cos(\eta)e^{i\phi_{N-1}} \\ \cos(\eta)e^{i\phi_N} & 0 & \cdots & 0 & 0 \end{pmatrix}$$

Eigenvalues and eigenvectors are found numerically from Eq. (S1). The propagation constants $\beta_j=\log(\lambda_j)/i$ versus mode number j define a dispersion curve.

3. Phase and intensities of the modes

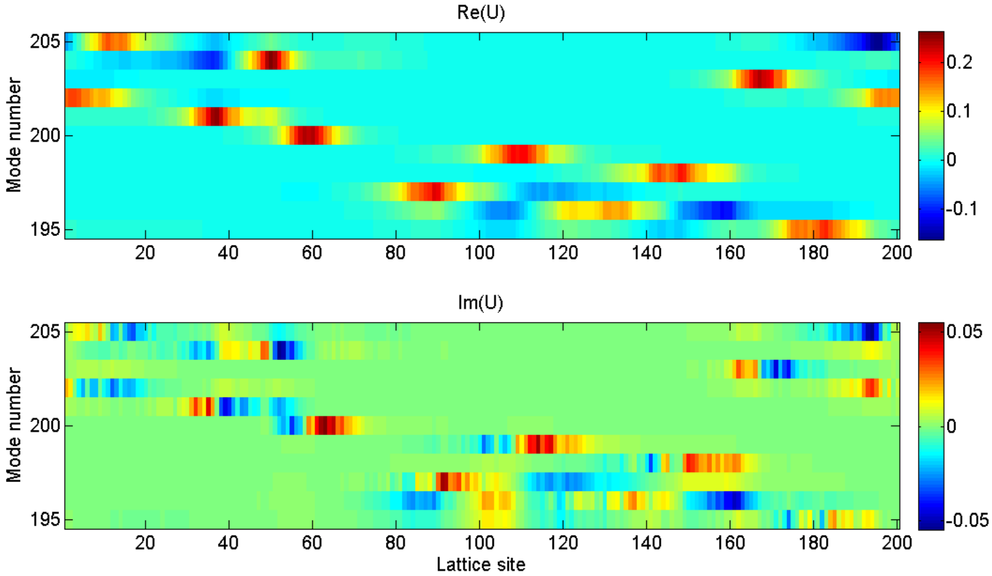

Figure S2. Eigenmode U_n real part (upper panel) and imaginary part (lower panel) as function of n, calculated for modes with numbers from 195 to 205. The level of disorder is ϕ_{max}=0.1 π and $\eta=\pi/4$.

To illustrate how intensity and phase of the modes U_n depend on the slot number n, we calculate the eigenmodes for a single realization of synthetic photonic lattice with disordered potential (Fig. S2). Potential strength is set to 0.1 π. We focus on the modes localized next to band gap.

We observe that each mode is localized at different groups of slots, as it can be seen from Fig. S2. A phase of the modes is slot dependent. Indeed, maximum of the real part of the mode does not coincide with the maximum of the imaginary part (compare upper and lower panels in Fig. S2). Then, it can be seen from smooth dependence Re(U(n)) (Fig.S2,upper panel) that phase is not staggered.

4. Numerical simulation of multiple-pulse initial excitation

Whereas in the manuscript we investigate the dynamics of single input pulses, here for comparison we study numerically the evolution of multiple pulses launched at the input (m=0). We find that individual localized modes are not excited more efficiently in this case compared with the case of single pulse excitation. The broad excitation results in complex evolution [Fig. S3] due to nontrivial inner structure of each mode. Indeed, initial flat-phased pulse train leads to an excitation of a large number of modes, as each of those has non-trivial inner phase.

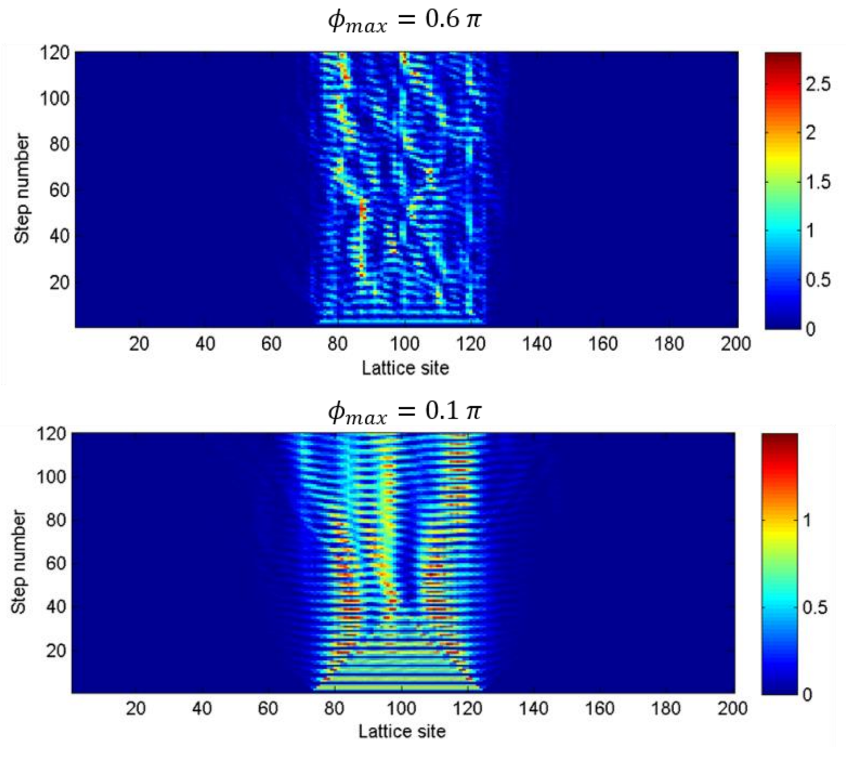

Figure S3. Numerical simulation of disordered synthetic photonic lattice excited with a broadband pulse train, with the disorder. Disorder strength is set to 0.6 π (upper panel) and $0.1\,\pi$ (lower panel).

References

1. M. Wimmer, A. Regensburger, C. Bersch, M.-A. Miri, S. Batz, G. Onishchukov, D. N. Christodoulides, and U. Peschel, "Optical diametric drive acceleration through action–reaction symmetry breaking," Nature Physics **9**, 780–784 (2013)
